# Supplementary material for: RNPS1 in PSAP complex controls periodic pre-mRNA splicing over the cell cycle
Source: iScience. 2024 Nov 15;27(12):111400. doi: 10.1016/j.isci.2024.111400 (PMC11648250; doi:10.1016/j.isci.2024.111400)
Supplement: Document S1. Figures S1–S6 and Table S1 [file mmc1.pdf]

iScience, Volume *27*

Supplemental information

RNPS1 in PSAP complex controls periodic pre-mRNA splicing over the cell cycle

Kazuhiro Fukumura, Akio Masuda, Jun-ichi Takeda, Osamu Nagano, Hideyuki Saya, Kinji Ohno, and Akila Mayeda

Table S1 List of all the synthetic oligonucleotides used in the experiments
(Related to STAR Methods)

**siRNAs (sense sequences) for cellular knockdown (*T*: deoxyribonucleotide)**		
RNPS1-siRNA	5′-GCAAAGACCGCUCAGAUGA*TT*-3′	Knockdown of *RNPS1*
ACIN1-siRNA	5′-GAGGCCUUCUGGAUUGACA*TT*-3′	Knockdown of *ACIN1*
PNN-siRNA	5′-GAAGGUAGACGCAUCGAAU*TT*-3′	Knockdown of *PNN*
SAP18-siRNA	5′-CCAGCGAGUUGCAGAUCUA*TT*-3′	Knockdown of *SAP18*
GPATCH8-siRNA	5′-GACCAGAGCUGCUAUAGUA*TT*-3′	Knockdown of *GPATCH8*
LUC7L3-siRNA	5′-GAAGUAGAACGUAGGAUCA*TT*-3′	Knockdown of *LUC7L3*
**Primer DNAs for plasmid constructions**		
AURKB/I4-E5-I5-F	5′-CAGTGTGCTGGAATTGTTCTGAGTTGCCTTCCTC-3′	Construction of pcDNA3-AURKB/E5-WT & pcDNA3-AURKB/E5-Δ#3
AURKB/I4-E5-I5-R	5′-TAGATGCATGCTCGAGGCTTCGGCTCAGGGGGC-3′	
AURKB/E5/inR-E6-F1	5′-GAAATCCAGGCCCACAAGTCCCAGATAGAG-3′	Construction of pcDNA3-AURKB/E5/inR-E6
AURKB/E5/inR-E6-R1	5′-CAACCCATACTGCAGGTGGGCCTGGATTTC-3′	
AURKB/E5/inR-E6-F2	5′-GAAATCCAGGCCCACCTGCAGTATGGGTTG-3′	
AURKB/E5/inR-E6-R2	5′-CTCTATCTGGGACTTGTGGGCCTGGATTTC-3′	
AURKB/E5/inL-E6-F1	5′-AAGAAAAGCCATTTCATAAGTCCCAGATAGAGAA-3′	Construction of pcDNA3-AURKB/E5/inL-E6
AURKB/E5/inL-E6-R1	5′-GGACCTTGAGCGCCACGGTGGGCCTGGATTTCGA-3′	
AURKB/E5/inL-E6-F2	5′-TCGAAATCCAGGCCCACCGTGGCGCTCAAGGTCC-3′	
AURKB/E5/inL-E6-R2	5′-TTCTCTATCTGGGACTTATGAAATGGCTTTTCTT-3′	
**Primer DNAs for assays**		
AURKB/E5-F	5′-GGAATTCCGGCGGCACTTCACAATTGATG-3′	Splicing assay of endogenous *AURKB* intron 5
AURKB/E6-R	5′-CCGCTCGAGGCTGTTCGCTGCTCGTCAA-3′	
MDM2/E8-F	5′-CTTTGATGAAAGCCTGGCTC-3′	Splicing assay of endogenous *MDM2* intron 9
MDM2/E10-R	5′-CCTGCCTGATACACAGTAACT-3′	
RNPS1-PCR-F	5′-GAAGCACATGGATGGAGGAC-3′	PCR & qPCR detections of endogenous *RNPS1* mRNA
RNPS1-PCR-R	5′-GGAGACCTGCGCCACATA-3′	
ACIN1-PCR-F	5′-GCAGACCAAGTCAGCAATGA-3′	PCR & qPCR detections of endogenous *ACIN1* mRNA
ACIN1-PCR-R	5′-CCCCCTCTGTGTCACTGTTT-3′	
PNN-PCR-F	5′-AGAAGAGAATCACGCCAGGA-3′	PCR & qPCR detections of endogenous *PNN* mRNA
PNN-PCR-R	5′-CCGCTTTTGCCTTTCAGTAG-3′	
SAP18-PCR-F	5′-ATGCTCGCTGCAGGGGTC-3′	qPCR detection of endogenous *SAP18* mRNA
SAP18-PCR-R	5′-TTAATATGGTCTCATGCGCCCT-3′	
GPATCH8-PCR-F	5′-CTTCAGGGTCTGCCTGTAGC-3′	PCR detection of endogenous *GPATCH8* mRNA
GPATCH8-PCR-R	5′-GTTTTCGTTTCTTGCGCTTC-3′	

LUC7L3-PCR-F	5′-CTCAAGCCGAACATCAGACA-3′	PCR detection of endogenous *LUC7L3* mRNA
LUC7L3-PCR-R	5′-CTCTGTCCCGACTTTTGCTC-3′	
ACTG1-PCR-F	5′-CCCATCTACGAGGGCTACG-3′	PCR & qPCR detections of endogenous *ACTG1* mRNA
ACTG1-PCR-R	5′-TTCATGAGGTAGTCGGTCAGG-3′	
AURKB-PCR-F	5′-CTCCTACCCCTGGCCCTA-3′	qPCR detection of endogenous *AURKB* mRNA
AURKB-PCR-R	5′-GATGGGGTGACAGGCTCTT-3′	
T7	5′-AATACGACTCACTATAG-3′	RIP assay for *AURKB*/E5-WT and *AURKB*/E5∆#3 reporter
AURKB/E6-R	5′-CCGCTCGAGGCTGTTCGCTGCTCGTCAA-3′	
ACTG1/E3-F	5′-TGACCCTGAAGTACCCCATT-3′	Splicing assay of endogenous *ACTG1* intron 3
ACTG1/E4-R	5′-TGGTGGTGAAGCTGTAGCCT-3′	
ADCK4/E6-F	5′-AATGCCGAGCGGATTGTG-3′	Splicing assay of endogenous *ADCK4* intron 6
ADCK/E7-R	5′-GGACCCGCTCAAAGATGTG-3′	
VPS13D/E44-F	5′-TCTCTTCACTCCCACCAGTG-3′	Splicing assay of endogenous *VPS13D* intron 44
VPS13D/E45-R	5′-GTGTCCATAGAGTGGCACTCT-3′	
HERC2/E61-F	5′-ACTGTGACAAACCAAGGCTG-3′	Splicing assay of endogenous *HERC2* intron 61
HERC2/E62-R	5′-CTTCATCGGTCAGAGCCAG-3′	
AURKB/spliced-F	5′-CAGGCCCACCTGCACCAT-3′	qPCR detection of endogenous *AURKB* spliced mRNA
AURKB/E6-R	5′-CCGCTCGAGGCTGTTCGCTGCTCGTCAA-3′	
AURKB/I5-F	5′-AGTGAGCCCCTTTAGCTCTT-3′	qPCR detection of endogenous *AURKB* intron 5 retained mRNA
AURKB/I5-R	5′-TGGGAGTGGTAAGGGGAAA-3′	
ADCK4/spliced-F	5′-AGATGCTCAGCATCCAGGAC-3′	qPCR detection of endogenous *ADCK4* spliced mRNA
ADCK4/E7-R	5′-GGACCCGCTCAAAGATGTG-3′	
ADCK4/E6-F	5′-AATGCCGAGCGGATTGTG-3′	qPCR detection of endogenous *ADCK4* intron 6 retained mRNA
ADCK4/I6-R	5′-GAGTCGAGGGTCATGCTGTA-3′	
VPS13D/spliced-F	5′-ATCAGCTCCAGACAAGCCAG-3′	qPCR detection of endogenous *VPS13D* spliced mRNA
VPS13D/E45-R	5′-GTGTCCATAGAGTGGCACTCT-3′	
VPS13D/I44-F	5′-GAGGACCATTGCCTGAAAGTG-3′	qPCR detection of endogenous *VPS13D* intron 44 retained mRNA
VPS13D/E45-R	5′-GTGTCCATAGAGTGGCACTCT-3′	
HERC2/E61-F	5′-ACTGTGACAAACCAAGGCTG-3′	qPCR detection of endogenous *HERC2* spliced mRNA
HERC2/spliced-R	5′-GACCGAGAAGGACTTTCACCA-3′	
HERC2/I61-F	5′-TGCCTGTGCGTGATTCTG-3′	qPCR detection of endogenous *HERC2* intron 61 retained mRNA
HERC2/E62-R	5′-CTTCATCGGTCAGAGCCAG-3′	

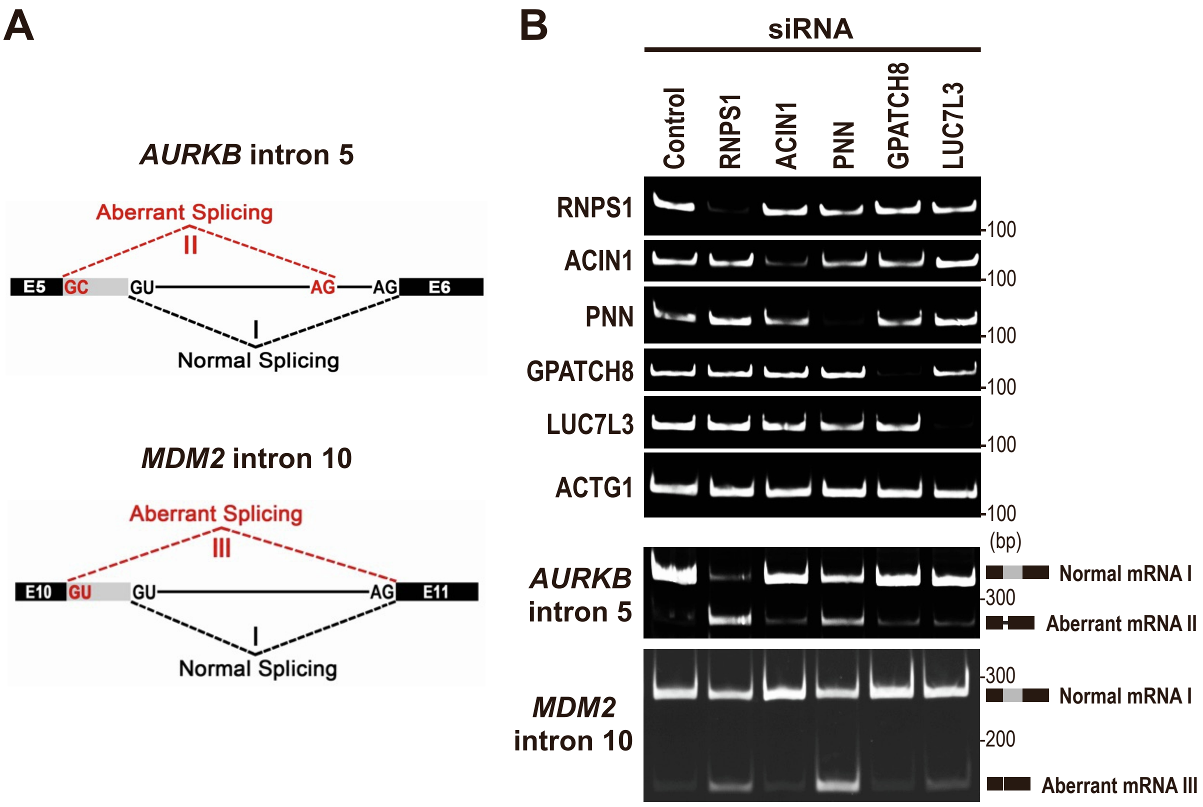

Figure S1. PSAP components, but not ASAP-specific component and other RNPS1-binding factors, promote normal splicing of two PSAP-controlled pre-mRNAs (Related to Figure 1C)

(A) Schematic diagram of pre-mRNA splicing pathways that generate the normal mRNA (I) and aberrant mRNAs (II, III) in the indicated pre-mRNA. Previously it was shown that these aberrant mRNAs (II, III) are generated by siRNA-mediated depletion of RNPS1, a component of of PSAP [S1].

(B) siRNA-mediated depletion of the ASAP-specific component (ACIN1), PSAP-specific component (PNN), and other RNPS1-binding proteins (GTATCH8 and LUC7L3) in HeLa cells and these proteins were analyzed by Western blotting with indicated antibodies (upper panel). Indicated spliced mRNAs (I, II, III) from the endogenous *AURKB* and *MDM2* genes were detected by RT–PCR and visualized by PAGE (lower panel). *GPATCH8* (G patch domain-containing protein 8) was isolated from human brain cDNA library and its function is unknown. Previous exome sequencing study showed that a mutation in *GPATCH8* associated with hyperuricemia co-segregating with osteogenesis imperfecta [S2]. *LUC7L3* (LUC7 like3) is a paralogues of yeast U1 snRNP component *LUC7*, which is involved in alternative splicing regulation in human [S3, S4].

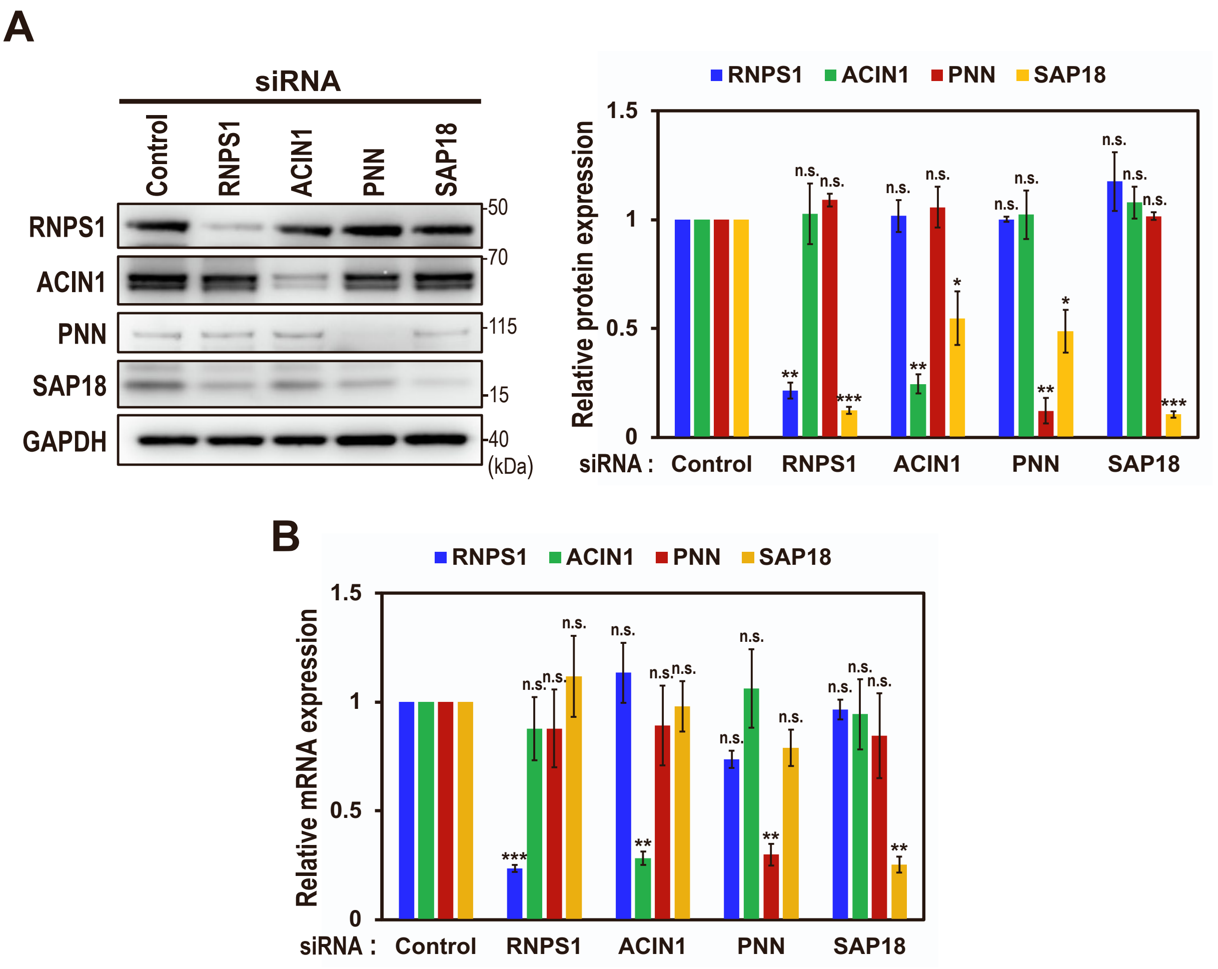

Figure S2. Knockdown of RNPS1, ACIN1 and PNN in HEK293 cells also caused co-depletion of SAP18 protein (Related to Figure 1A, B)

(A) siRNA-mediated depletion of the indicated ASAP/PSAP components in HEK293 cells were analyzed by Western blotting with indicated antibodies (left panel). Individual bands on the Western blots were quantified and the relative values were standardized to that in the control siRNA (right graph). Means ± standard errors (SE) are given for three independent experiments and Welch's t-test values were calculated (*p < 0.05, **p < 0.005, ***p < 0.0005, n.s. p > 0.05).

(B) The mRNA levels of ASAP/PSAP components in (A) were analyzed by RT-qPCR using specific primer sets. See (A) for the statistical analysis.

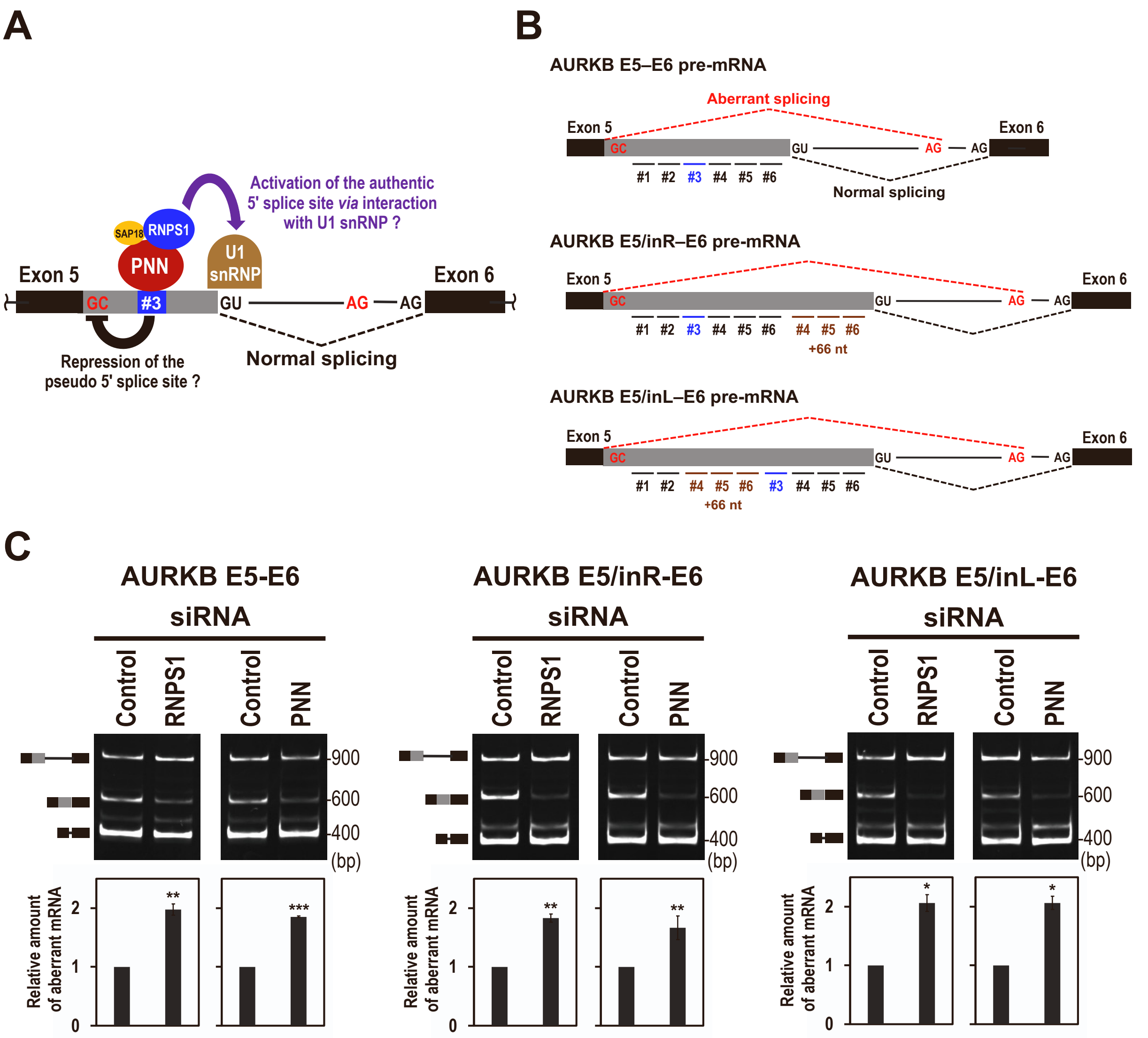

Figure S3. PSAP either represses the upstream pseudo 5′ splice site or activates the downstream authentic 5′ splice site to ensure precise splicing of *AURKB* pre-mRNA (Related to Figure 1C)

(A) Schematic diagram of two conceivable PSAP-mediated mechanisms to maintain normal splicing. (i) PSAP bound on the target site (#3) represses the upstream pseudo 5’ splice site through either direct masking or indirect interference. (ii) PSAP on the target site (#3) activates the downstream authentic 5’ splice site through RNPS1 interaction with a component of U1 snRNP [S1].

(B) Three *AURKB* mini-gene transcripts to test the above models. The distances from PSAP-binding site (#3) to either upstream authentic 5’ splice site or downstream pseudo 5’ splice site were expanded by inserted duplicated fragments (#4-#5-#6) in E5/inR–E6 and E5/inL–E6 mini-genes, respectively.

(C) *In cellulo* splicing assay of ectopically expressed these three *AURKB* mini-genes upon depletion of PSAP components, RNPS1 and PNN. Splicing products were detected by RT–PCR, visualized by PAGE, and individual bands on the PAGE gel were quantified. See the legend to Figure 1C for the methods of quantification and statistical analysis.

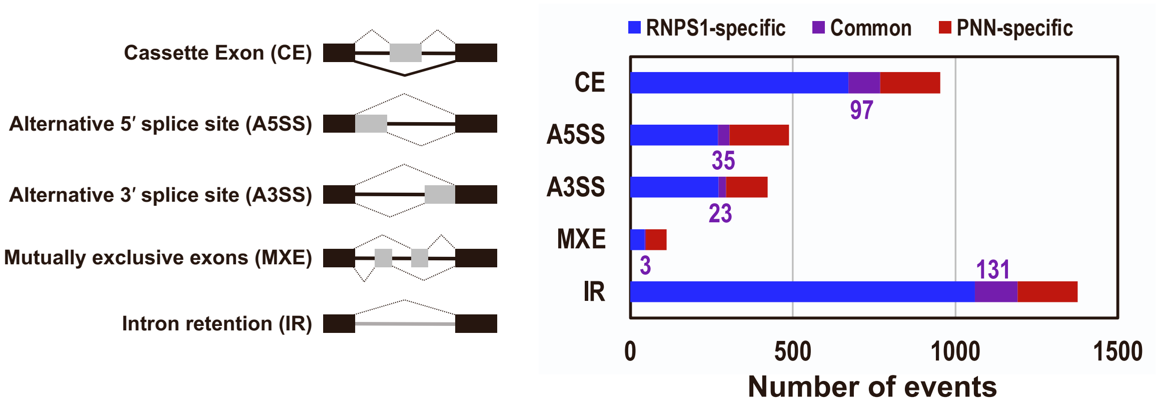

Figure S4. Knockdown of PSAP components, RNPS1 and PNN, causes global changes in splicing patterns (Related to Figure 2A)

RNA-Seq analyses of RNPS1- and PNN-knockdown HEK293 cells were performed. The number of statistically significant changes in each alternative splicing pattern (left panel) is visualized as bar graph (right graph). The purple numbers show the common splicing changes, in either directions, induced in RNPS1- and PNN-knockdown HEK293 cells.

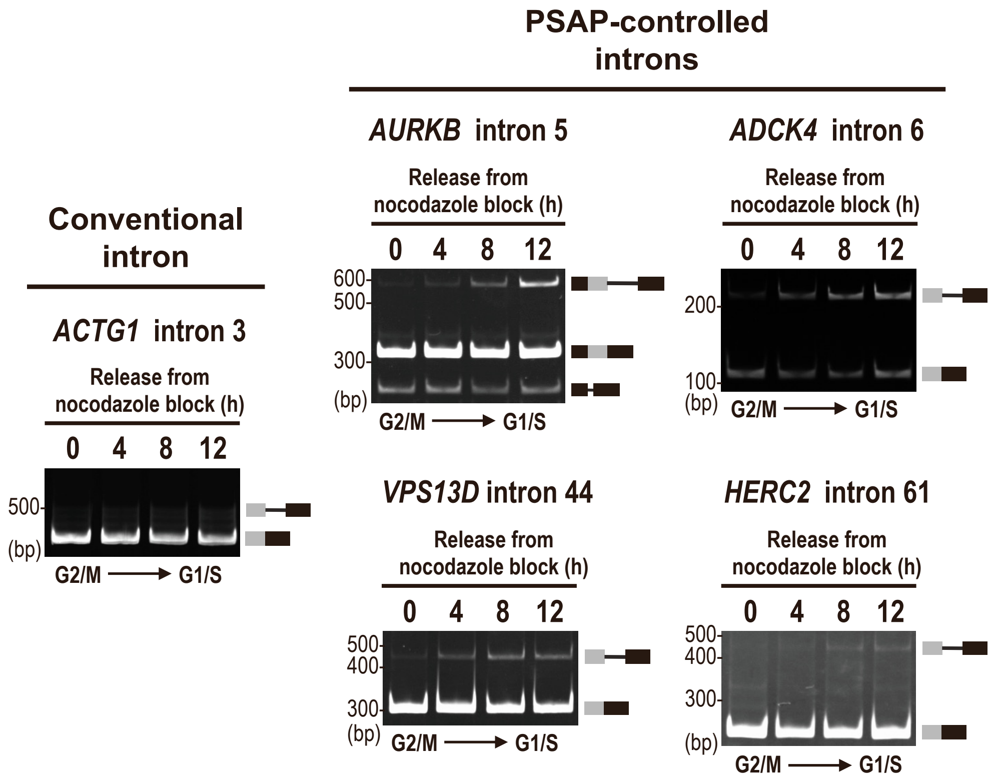

Figure S5. Periodic generation of unspliced introns from G2/M to G1/S phase were observed in PSAP-controlled introns but not in conventional intron (Related to Figures 3B, 4)

HEK293 cells were synchronized in G2/M phase by nocodazole block. Endogenous splicing in the indicated conventional intron and PSAP-controlled introns was analyzed by RT-PCR followed by PAGE.

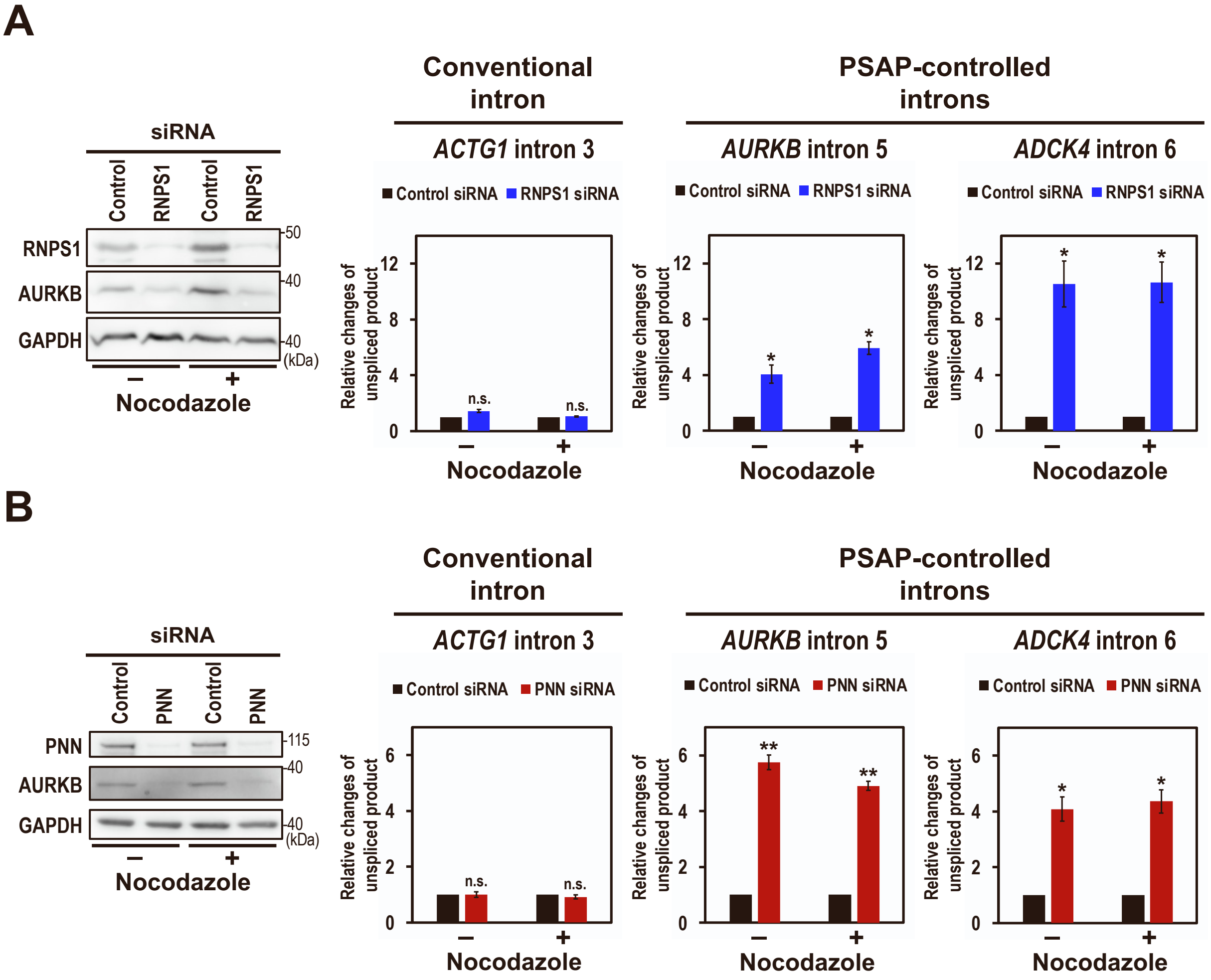

Figure S6. Knockdown of RNPS1 and PNN generates unspliced products in PSAP-regulated introns, but not in conventional intron, irrespective of cell cycle phase (Related to Figures 3–5)

(A) RNPS1 was depleted by siRNA-mediated knockdown in HEK293 cells and treated with nocodazole to arrest the cell cycle at the G2/M phase. Proteins were analyzed by Western blotting with indicated antibodies. *AURKB* is downregulated by RNPS1-knockdown as previously described [S1]. Indicated retained introns were analyzed by RT–qPCR and the values were standardized to that of the control-knockdown and plotted. Means ± SE are given for three independent experiments and Welch's t test values were calculated (*$p < 0.05$, n.s. $p > 0.05$).

(B) PNN was depleted by siRNA-mediated knockdown in HEK293 cells and treated with nocodazole to arrest the cell cycle at the G2/M phase. The same analyses as in (A) were performed. See (A) for the statistical analysis (*$p < 0.05$, **$p < 0.005$, n.s. $p > 0.05$).

SUPPLEMENTAL REFERENCES

S1. Fukumura, K., Inoue, K., and Mayeda, A. (2018). Splicing activator RNPS1 suppresses errors in pre-mRNA splicing: A key factor for mRNA quality control. Biochem. Biophys. Res. Commun. *496*, 921–926. https://doi.org/10.1016/j.bbrc.2018.01.120.

S2. Kaneko, H., Kitoh, H., Matsuura, T., Masuda, A., Ito, M., Mottes, M., Rauch, F., Ishiguro, N., and Ohno, K. (2011). Hyperuricemia cosegregating with osteogenesis imperfecta is associated with a mutation in GPATCH8. Hum. Genet. *130*, 671–683. https://doi.org/10.1007/s00439-011-1006-9.

S3. Fortes, P., Bilbao-Cortes, D., Fornerod, M., Rigaut, G., Raymond, W., Seraphin, B., and Mattaj, I.W. (1999). Luc7p, a novel yeast U1 snRNP protein with a role in 5' splice site recognition. Genes Dev. *13*, 2425–2438. https://doi.org/10.1101/gad.13.18.2425.

S4. Gao, G., Xie, A., Huang, S.C., Zhou, A., Zhang, J., Herman, A.M., Ghassemzadeh, S., Jeong, E.M., Kasturirangan, S., Raicu, M., *et al*. (2011). Role of RBM25/LUC7L3 in abnormal cardiac sodium channel splicing regulation in human heart failure. Circulation *124*, 1124–1131. https://doi.org/10.1161/CIRCULATIONAHA.111.044495.
